# Short-range homing in camels: displacement experiments

**DOI:** 10.1242/bio.058850

**Published:** 2021-08-06

**Authors:** Sofyan H. Alyan

**Affiliations:** Department of Biology, United Arab Emirates University, P.O. Box 15551, Al Ain, United Arab Emirates

**Keywords:** *Camelus dromedaries*, Homing, Navigation, Path integration

## Abstract

Camels (*Camelus dromedarius*) are known to have good navigational abilities and can find their home after displacement to far places; however, there are no studies available on the navigational strategies employed by the camels in homing behavior. Thus, the aim of this study was to investigate these strategies by displacing female camels equipped with GPS trackers 6 km away from home to totally unfamiliar locations. The experiments comprised displacing nursing or non-nursing female camels 6 km from their living pens to an unfamiliar release site. Some camels were taken to the release site on foot, others were hauled on a truck, both during daytime and nighttime. Displacements journeys were either in a straight direction to the release points, or they consisted of a convoluted path. As a result, camels that had straight outward journeys were able to return home efficiently and rather directly, but camels that had convoluted trips to the release point failed to do so. Moreover, impairing olfactory, visual, and auditory inputs by using mouth/nose muzzles, eye covers and headphones did not affect homing ability. Based on these experiments the most likely hypothesis is that during their small-scale round trips the camels relied on path integration, and that this strategy is disrupted when the camels were subjected to disorientation procedures before release.

## Abstract (precis)

**Summary:** The study reports a series of experiments aimed at understanding the orientation mechanisms of Arabian camels in the Rub' al Khali desert in the UAE. Camels were taken either on foot or inside trucks to unfamiliar release points, some 6 km from their living pens. Camels homed successfully after simple displacements but seemed lost after a looping journey. It is inferred that camels use path integration, dead reckoning, after short simple displacements.

## INTRODUCTION

Camels (*Camelus dromedarius*) are a hallmark of the Arabian Desert. There have been numerous studies on camels, including those on adaptations, physiology, anatomy, immunology, morphology, nutrition, and several other aspects related to camel health and utility for humans (Gebreyohanes and Assen, 2017; Root-Bernstein and Svenning, 2016; Vyas et al., 2014; Yam and Khomeiri, 2015). In contrast, there has been a remarkable paucity of research on the natural, wild behavior of camels (Lethbridge et al., 2010). Even though camels are known as ‘desert ships’, their navigational abilities have not yet been studied. A camel owner told me, “I had camels transferred from my farm near Omani borders to an island off Abu Dhabi. Few days later, the camels went missing and were later found in the old farm. They swam and travelled all the way to get there”. Despite the anecdotes on the amazing ability of camels to cross vast stretches of deserts, the scientific literature is void of detailed studies on how the camels accomplish these tasks.

Over more than half a century, various taxonomies of navigational strategies have been proposed ranging from homing by beacons, to performing path integration, and finally to using cognitive maps. These strategies have been investigated in many groups of animals, particularly in arthropods, turtles, birds, and mammals (Alyan and Jander, 1994; Fuxiage et al., 2011, Gallistel, 1990; Able, 2000; Trullier et al., 1997; Benhamou, 2010; Wiener et al., 2011; Wiltschko and Wiltschko, 2015; Åkesson et al., 2015; Warren, 2019; Wehner, 2020). However, none of these navigational strategies have been examined in camels.

In this respect, the paucity of detailed studies applies to other large terrestrial mammals as well. Some studies deal with the behavior of animals along migration routes, e.g. studies performed in polar bears (Rogers, 1986), caribous, wapitis, and mule deer (Ramsay and Andriashek, 1986; Craighead and Craighead, 1987; Morgantini and Hudson, 1988; Thomas and Irby, 1990), while in others round-trip excursion routes have been recorded, e.g. in polar bears (Rogers, 1987a), or homing successes of displaced individuals have been determined, e.g. in black bears (Rogers, 1986). In general, experimental translocations of some species of large mammals revealed that carnivores are more likely to home successfully than herbivores (Rogers, 1984, 1988). According to a recent study, elephants seem to use habitual routes when travelling in the less familiar periphery of their home range area, while resorting to a cognitive map when moving within the core area (Presotto et al., 2019). The use of Euclidian cognitive maps has especially been proposed in wild chimpanzees (Boesch and Boesch, 1984).

In the current study, displacement experiments are used to examine how female camels navigate over short distances (less than 10 km), whether they can do so by path integration, and whether there are differences between nursing and non-nursing females.

## RESULTS

### Controls

#### Control 1

**Fig. 1.**
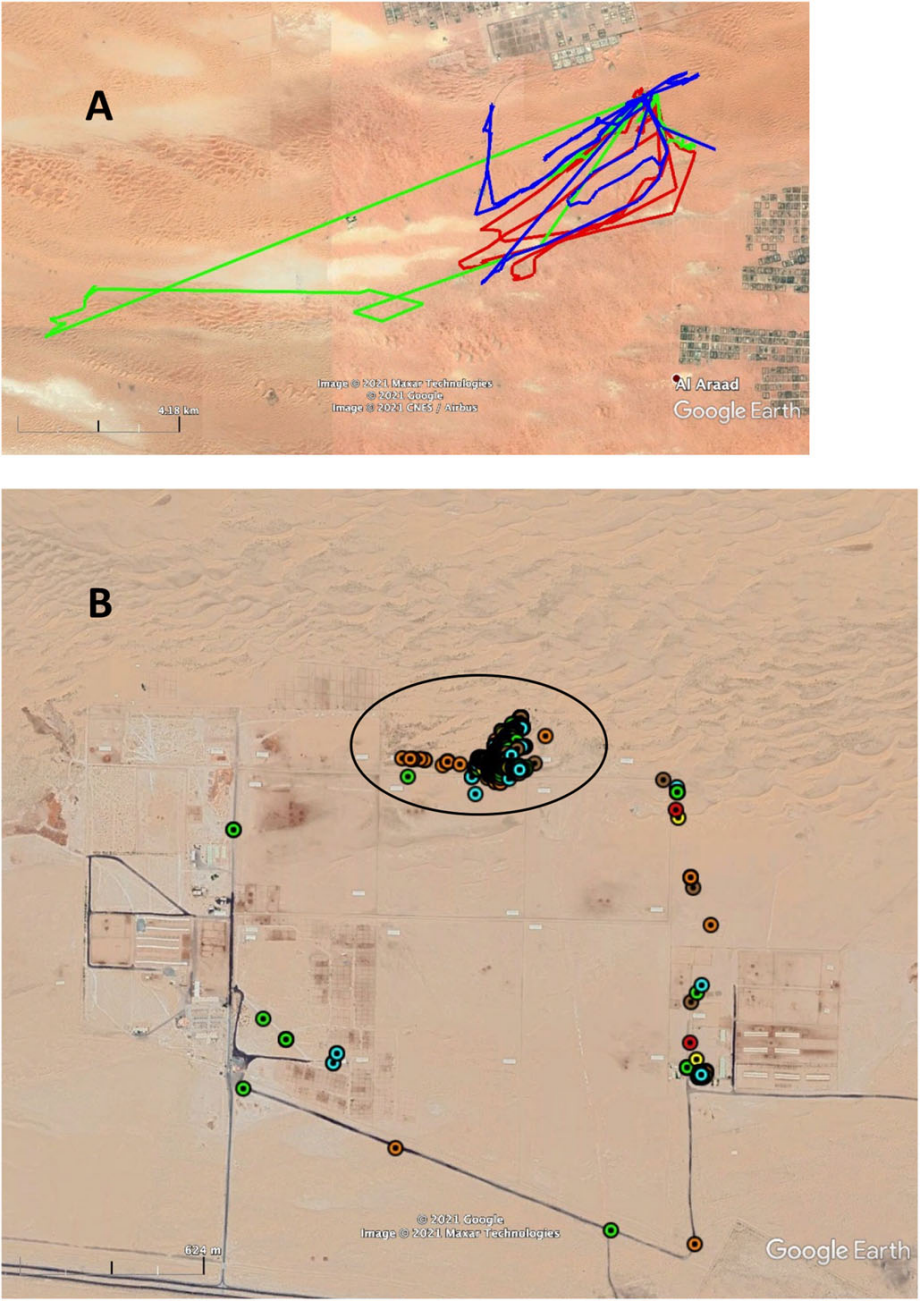
**Comparison between free-ranging camels and movement-restricted camels.** (A) Tracks of three free-ranging camels that moved back and forth to their farm after one (shortest loops) or a few days (longest loops). (B) Camels that were used for the current experiments were moved from their enclosures to another that is enclosed in the black oval. Positions of six camels are shown in this figure.

Control 1 comprised monitoring four female camels for 1–4 days on a farm where camels could freely range far from home. Sometimes, the camels went back and forth to the farm every day, when at other times they travelled in expeditions lasting from a few days to weeks. The goal of this control was to show that camels exhibit homing behavior when they freely range on their own. Fig. 1A shows some of the tracks of three free-ranging camels.

#### Control 2

Control 2 comprised monitoring of camel movements in the ASG husbandries before the experimental treatments. The camels did not leave their husbandries to forage in the surrounding areas. Fig. 1B shows the GPS tracks of six camels over 3 days.

### Experiment 1: on foot

Camels were led on foot from their quarters to either the eastern release point (ERP, seven animals) or western release point (WRP, three animals). The shepherd drove an all-terrain vehicle (ATV) while securing the camel with a rope. The outward journeys took place during daytime or nighttime. During each journey, one camel was taken. Sometimes, two camels were taken on separate journeys on the same day, depending on the availability of the shepherd. Camels were released and free to move on their own. Five camels were used during daytime runs, and another five were used during nighttime runs, thus using a total of ten camels. Of those, six were non-lactating and four were lactating.

**Fig. 2.**
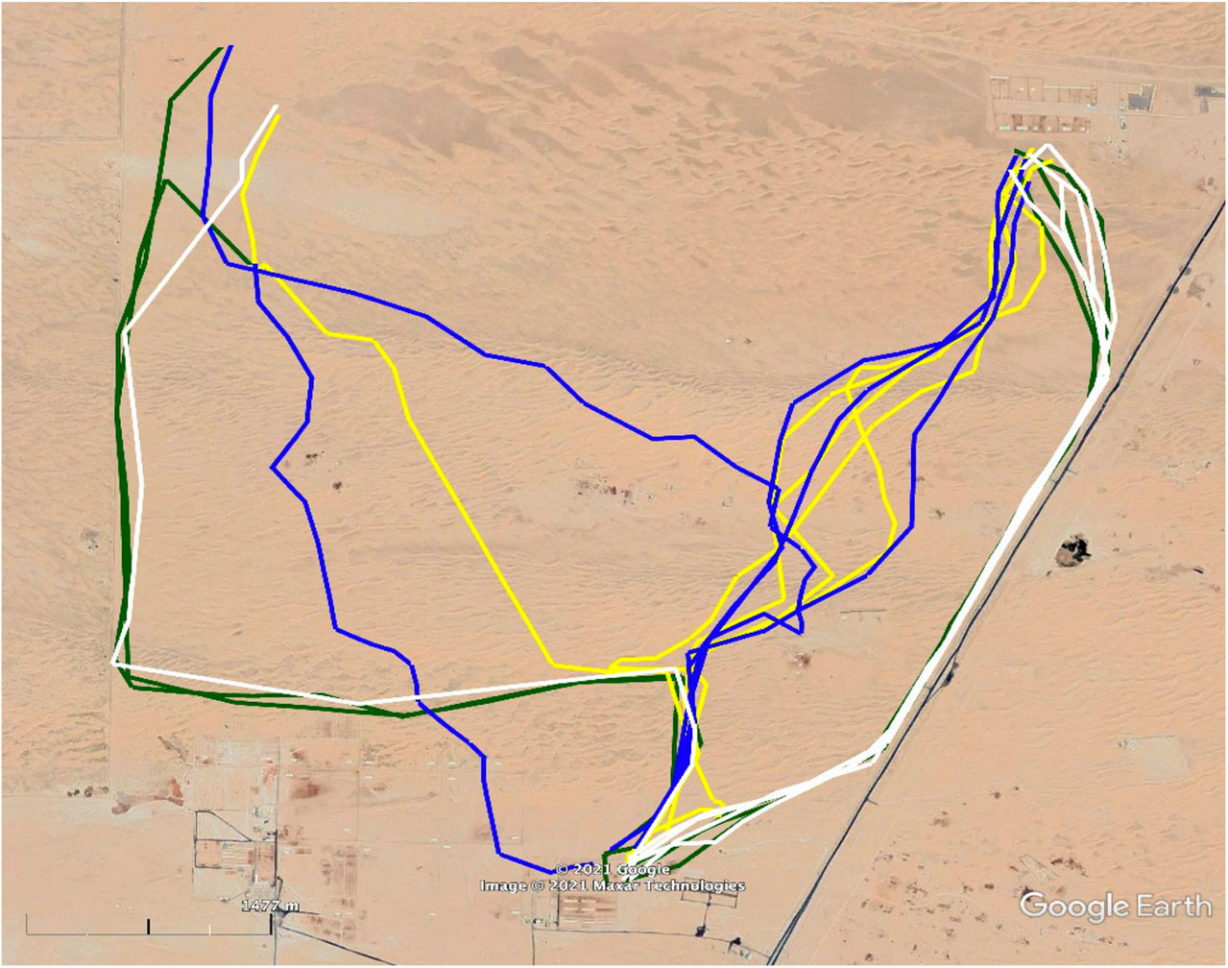
**Experiment 1.** GPS tracks of outward and homeward journeys of all camels taken on foot from the ASG husbandries to the ERPs and WRPs, respectively. White lines indicate nighttime outward paths. Yellow lines indicate nighttime homeward paths. Green lines indicate daytime outward paths. Blue lines indicate daytime homeward paths.

**Fig. 3.**
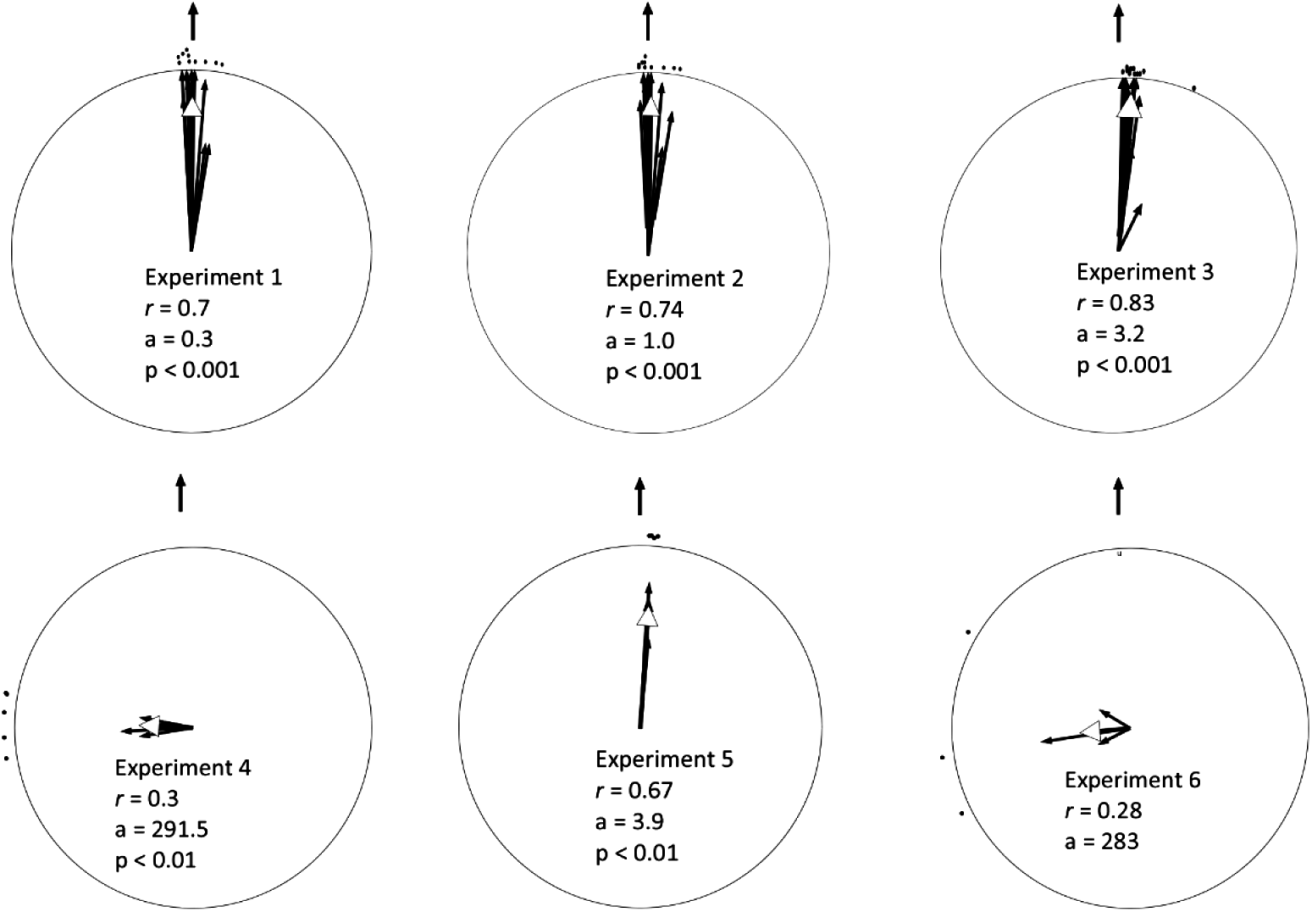
**Individual mean homing vectors of each camel in the different experiments (individual black arrows) and the mean vector (white arrow) length, angle, and significance level for each experimental set.** The vector length is proportional to the radius of the circle. Home direction is indicated by the black arrow outside the circle.

Different camels were taken on foot to two release sites. The tracks of the outward and homeward journey are depicted in Fig. 2. The mean vector of this group of camels was significantly different from random (Hotelling test, *r*=0.7, *α*=0.3, *P*<0.001; Fig. 3). The homing efficiency index (EI) data are shown in Table 1. Durations of outward and homeward trips are shown in Table S2.

**Table 1.**
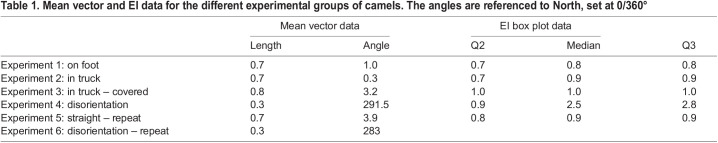
Mean vector and EI data for the different experimental groups of camels. The angles are referenced to North, set at 0/360°

#### Experiment 2: in truck

The camel was guided into the truck. The truck driver drove to the third release point (3rdRP). The outward journey took place during daytime or nighttime. For each journey, one camel was taken. Sometimes, two camels were taken on separate journeys during the same day, depending on the availability of the shepherd. Three camels were used for daytime runs, and seven camels were used for nighttime runs, making a total of four non-lactating and six lactating camels.

**Fig. 4.**
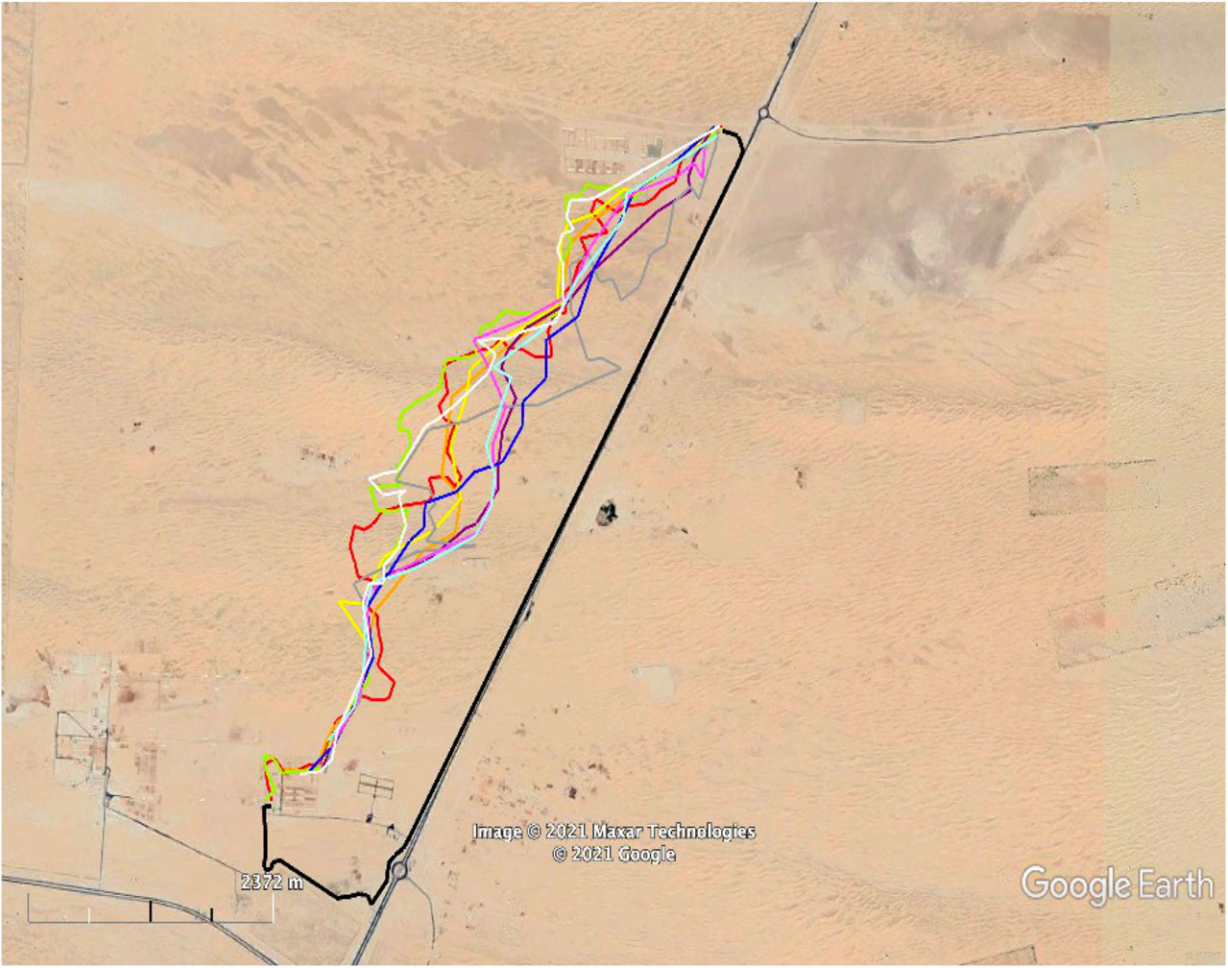
**Outward track (black line) and homeward tracks (colored lines) of the camels in experiment 2.** The blue, red and grey lines indicate daytime releases. Homing vector angle and length relative to home is 199° and 7 km, respectively.

Fig. 4 depicts the outward and homeward tracks of the ten camels that were taken in a truck to the 3rdRP. The mean vector of this group of camels was significantly different from random (Hotelling test, *r*=0. 7, *α*=1.0, *P*<0.001; Fig. 3). The homing EI data are shown in Table 1.

#### Experiment 3: in truck – covered up

**Fig. 5.**
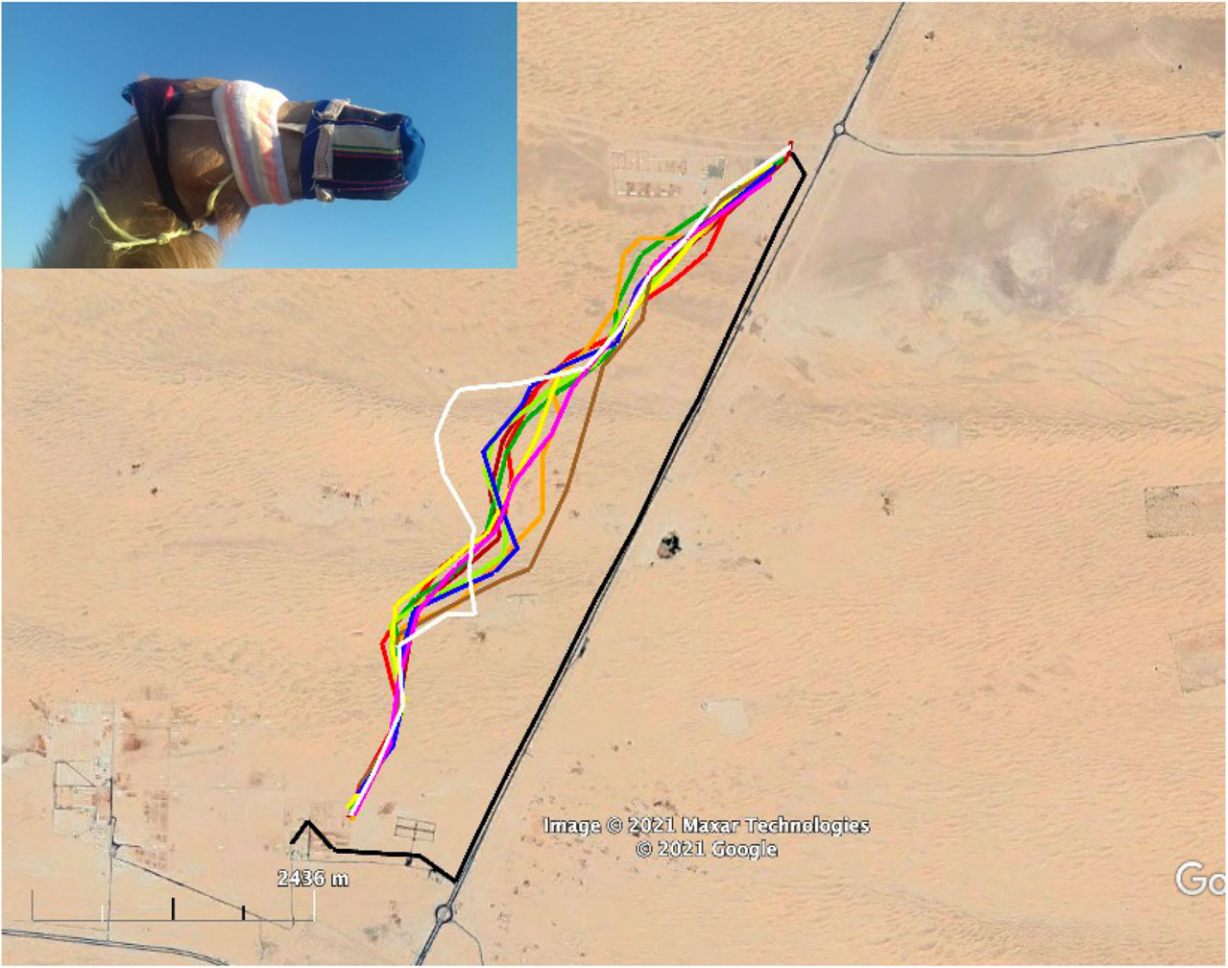
**Outward (black line) and homeward tracks (colored lines) of camels that were taken in a truck to the 3rdRP.** The camels had their eyes, noses and ears covered during the outward journey. During the homeward journey, the camels had only their nose and ears covered (inset). The red, orange, brown, burgundy, and white lines indicate daytime runs.

Camels were taken following a similar procedure as that used for the previous experiment. However, a camel muzzle (a so-called letham) was placed on the mouth/nose area. The muzzle had four pockets on the sides and one in the front. Two side pockets were filled with activated charcoal and baking soda to minimize the outside odors when the camel was breathing. The other two side pockets and front pocket were filled with natural aromatic spices or seeds [lavender, cinnamon, chamomile, cardamom and kheil (Arabic for ferula, which has a strong, pungent smell)]. In addition, a long scarf was used to cover the eyes, and headphones (3M Peltor X-Series Over-the-Head Earmuffs, NRR 31dB) with a scarf wrapped around the headphones to cover the ears. These control measures were used to minimize environmental cues that might give the camel a sense of direction on the outbound journey (Fig. 5, inset). This, however, would not exclude visual guidance during the homeward journey.

The shepherd placed the covers after the camel had boarded the truck. The eye cover was removed before the camel left the truck at the 3rdRP, but the nose and ear covers were left in place. Table 1 shows the number and status of all females used in this experiment.

The camels' tracks are shown in Fig. 5. The mean vector of animals' directional choice is significantly different from random (Hotelling test, *r*=0.8, *α*=3.2, *P*<0.001; Fig. 3). The homing EI data are shown in Table 1.

#### Experiment 4: in truck – disorientation – naïve

In this experiment, the camels were taken in a truck to a model-farm area 11 km away from the ASG quarters. The truck drove in many loops around the farms, before heading to the 3rdRP.

**Fig. 6.**
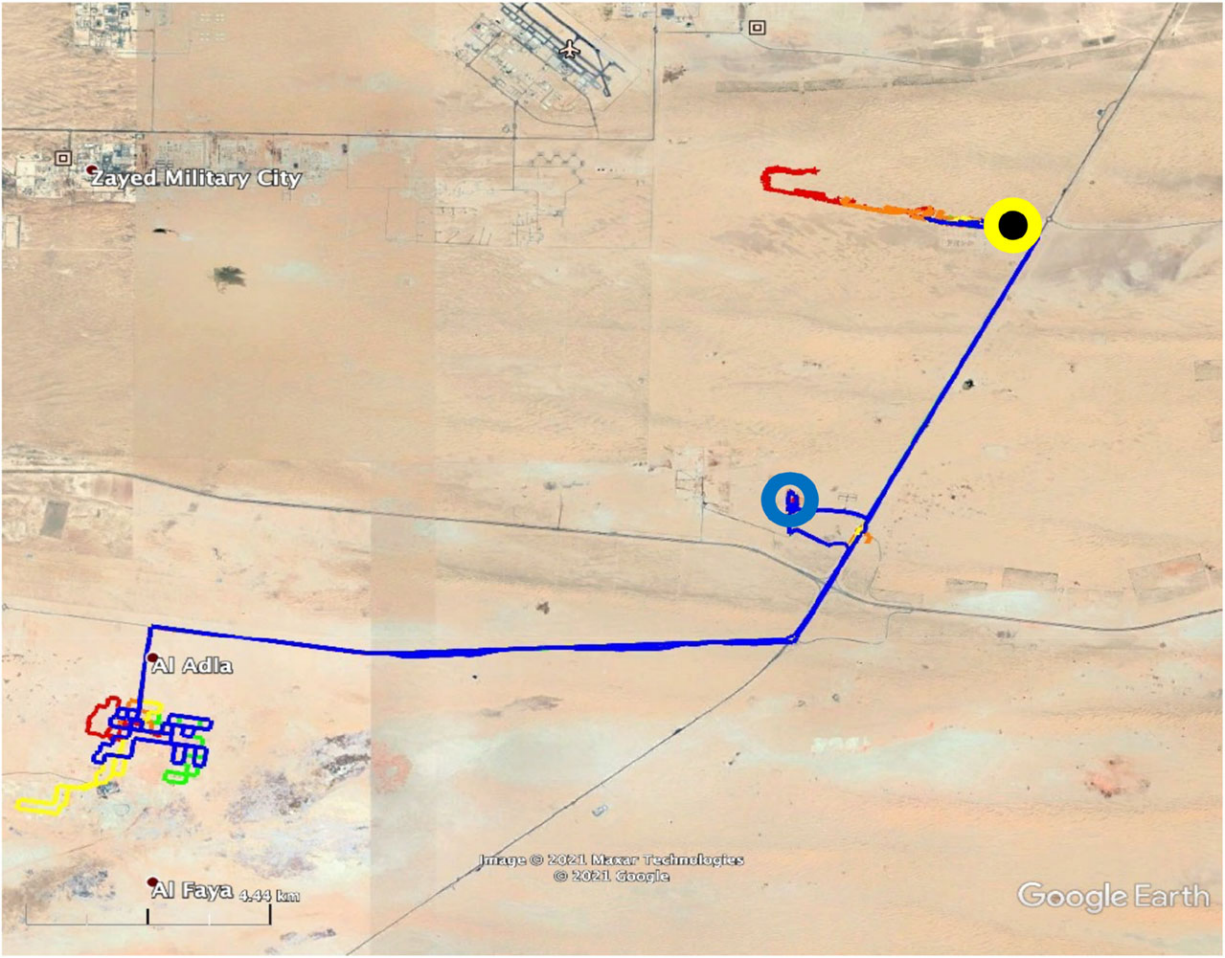
**The paths taken by five camels subjected to disorientation procedures.** All camels start from home, indicated by the blue circle, while carried inside a truck. Truck departed home, sometimes in different directions. The 3rdRP is the black circle with a yellow perimeter in the upper right corner. All camels headed in a westerly direction and spent the night in that area. Camels were transported on the next day back to the ASG quarters in trucks.

The homing tracks in this experiment differed substantially from the ones used in all previous experiments. All camels took a westerly direction and spent the remainder of the day at a random location they had ended up for that day. The next day, GPS trackers were used to determine the exact locations of the camels, after which the camels were returned to the ASG husbandries. The total journey of the five camels can be seen in Fig. 6. Two camels were taken at night, and three camels were taken during the daytime. Only one female was lactating. The mean vector of this group of camels was significantly different from random, although the direction of the vector was approximately 90° from home (Hotelling test, *r*=0.3, *α*=291.5, *P*<0.01; Fig. 3). The homing EI data are shown in Table 1.

#### Experiment 5: in truck – straight – repeat

This experiment was performed in retrospect after analyzing data of experiment 4. The four camels, three non-lactating and one lactating, used for this experiment were from a group that had been subjected to disorientation during the outbound journey. However, in this experiment, each of the four camels was taken in a truck, during daytime, in a straight path to the 3rdRP as in experiment 2.

**Fig. 7.**
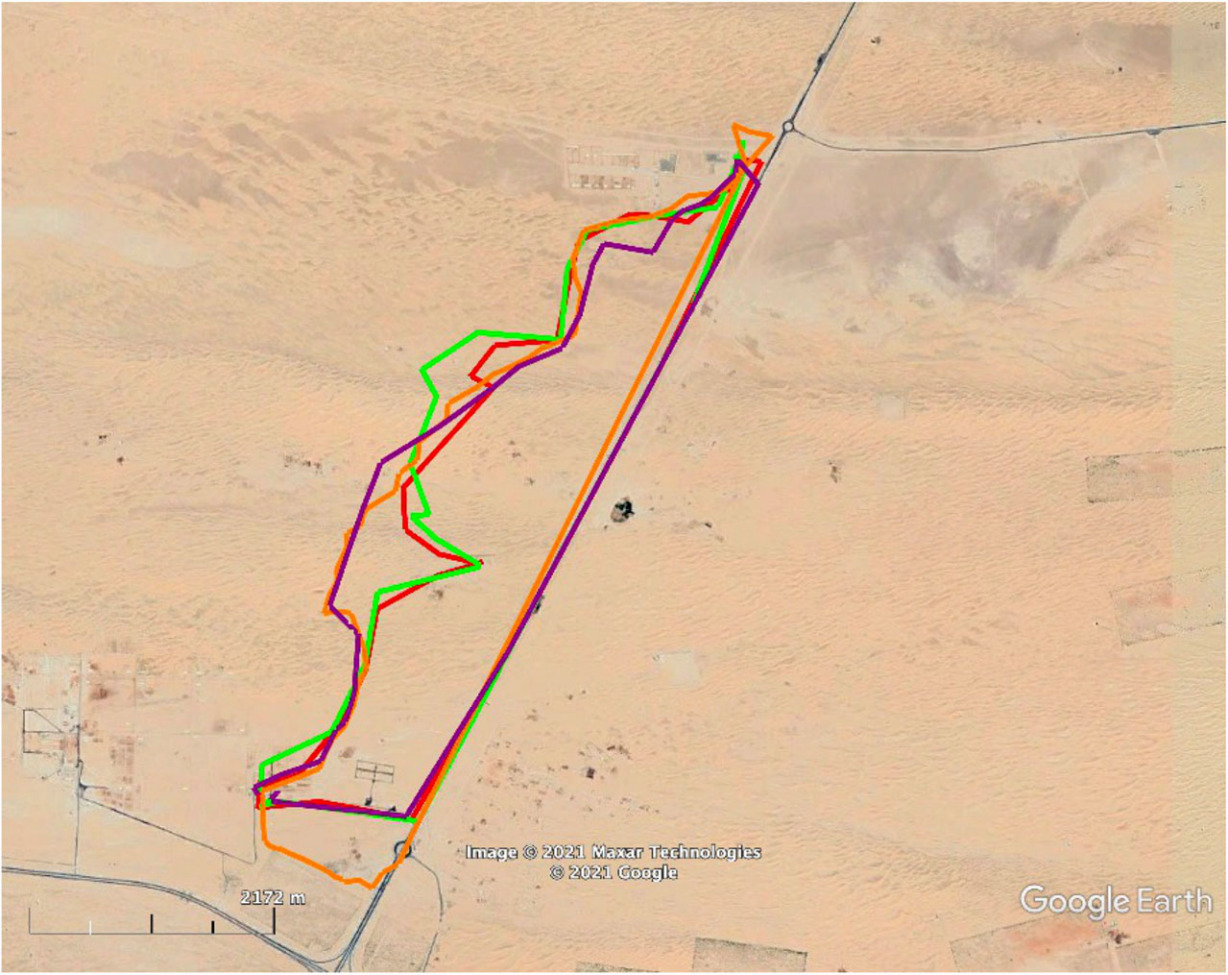
**Four camels that were subjected to disorientation during a previous outward journey were taken to the 3rdRP in a straight journey.** All four camels returned home, as shown in the tracks.

The four camels returned to the ASG quarters (Fig. 7). The mean vector of this group of camels was significantly different from random (Hotelling test, *r*=0.7, *α*=3.9, *P*<0.01; Fig. 3). The homing EI data are shown in Table 1.

#### Experiment 6: in truck – disorientation – repeat

This experiment was performed in retrospect, after considering the data on the disorientation experiment. The same disorientation procedure was followed for three camels that had already been used in experiment 2 above. This experiment was performed to ensure that the results obtained for camels in the preceding experiment were contingent on the disorientation of the outward path. Three camels were used, two of which were lactating, and one was non-lactating. We could not use any more camels because the rest of the camels were fertilized at that time. Two runs were performed at night, and one was performed during daytime.

**Fig. 8.**
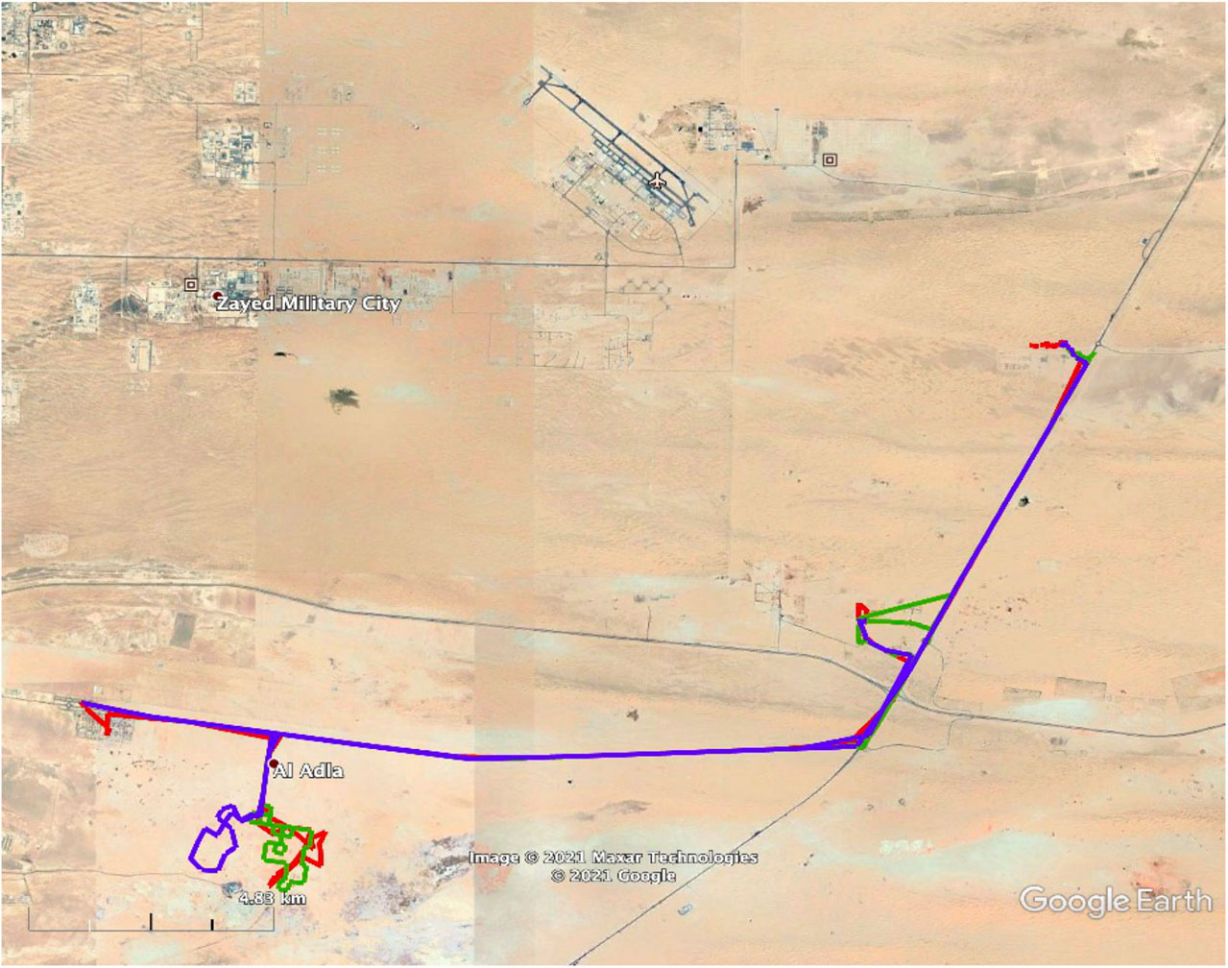
**This is a repeat experiment in which camels that were used in experiment 2, and showed good homing performance, were taken on a disorientation trip.** The camels could not return home after the looping excursion.

Fig. 8 shows the camels going off in the same westerly direction after being taken on the disorientation trip. Camels were picked up the next morning and taken back to ASG quarters. Owing to the small sample size, no statistical analysis was performed for this group of camels (*r*=0.3, *α*=84.8, Fig. 3).

## DISCUSSION

Several experiments were conducted to investigate the homing behavior of camels. Naïve camels were taken from their living pens to a new location that was 5–6 km away. Camels were taken to the release location on foot or were transported in a truck. The results can be summarized as follows. First, freely ranging domestic camels raised on farms learn to go back to their home after spending time foraging away from it. Second, camels that were displaced to a non-familiar location 6 km away from home, returned, regardless of whether this displacement was active (guided on foot) or passive (transported in a truck). In the latter case, their homing performance was not affected when their sight, smell and hearing were impaired on the way out, the homing trip was performed during daytime or nighttime or by suckling status. However, efficiency was impaired when camels were subjected to disorientation during outward passive transport. So, camels seemed to rely on path integration to infer the general direction of home. However, visual or other guiding cues were employed at different instances while proceeding towards home.

The main question of this study was to determine how camels find their way back home after being displaced by few kilometers. Three mechanisms could be used to achieve this task: guided orientation, orientation by path integration and orientation by learned landmark constellations (Åkesson et al., 2015). The camels that we used were unfamiliar with the release area, and the release point was separated from the homing pens by high sand dunes. This minimized the reliance on visual guiding cues or visual landmarks to find their way home. Additional support for this is that releases were made during daytime and nighttime, and the camels homed well under both conditions. However, this method did not preclude the use of olfactory, auditory, or other cues to find home. These cues could be used as guidance or as the basis for a cognitive mapping system. Despite these limitations, the findings increased the probability that the camels relied on path integration to find their way back home. The guiding cues and landmark orientation strategies were further weakened as possibilities because camels were masked during passive transport to the release area. Thus, gathering any such information about their surroundings would have been impossible without normal function of essential sense modalities during transport. The disorientation experiment further confirmed that the camels relied mainly on a path integration strategy to find their home. Camels that were transported in trucks and taken along a looping winding path were unable to find their home; they had to be picked up from their last stop and returned to the homing quarters by truck. These same camels homed well when they were carried in a truck to the release site in a simple, straight outbound path. Conversely, camels that previously homed after a straight displacement could not find their way home after a convoluted transport. It is important to mention here that the camels that were subjected to the disorientation procedure did not seem to be stressed out when examined by veterinarians, when taken back to the ASG quarters. A guidance mechanism would not have been impaired by such a looping trip or a landmark strategy, whether based on visual, olfactory, or magnetic cues emanating from the home pens. Furthermore, because the distances travelled during the disorientation trip were approximately 22 km back and forth, it seems plausible to propose that path integration might be used by camels for homing over short-range distances, which is mediated by idiothetic cues, such as employing their vestibular system for sensing linear and angular accelerations, as it has been described for small mammals (Mittelstaedt and Mittelstaedt, 1980; Etienne, 1980; Séguinot et al., 1988; Etienne et al., 1998; Etienne and Jeffery, 2004). Path integration via idiothetic cues would result in error accumulation in estimating home direction relatively quickly (Heinze et al., 2018; Wehner, 2020). However, idiothetic path integration is especially vulnerable to the accumulation of internally and externally generated noise, and hence render it an unlikely strategy for long-distance navigation (Mittelstaedt and Glasauer, 1991; Cheung et al., 2007, 2008).

It is important to emphasize that the proposition here that path integration is hypothesized to be the general mechanism used for navigation over 6–7 km distance does not exclude using environmental features, whether visual, olfactory, or magnetic cues, for arriving at home. For example, it can be seen from Fig. S6 that all camels seem to converge to a specific route towards the end of their homing trip. In this particular instance, dirt roads seem to be used by the camels while moving. In addition, lights coming from big cities surrounding the study area may well be used for orienting in a specific direction towards home. Attraction to specific features, like small farm remains, or the presence of other camels that might have been wandering in the area can also explain the long stretches and sharp turns that are taken by the camels in some cases.

Could geomagnetic cues have been contributed instead (Kimchi et al., 2004)? Further experimental research on the camels' amazing homing behavior should deal with this question, and particularly extend the approach applied in the present study to larger displacement distances. It is also important to mention here that any compass reference, such as lights, stars, moon, and magnetic cues, and an odometer may have been used instead of idiothetic cues, mentioned above, to calculate the vector towards home. Given the paucity of information about navigational strategies in large cursorial terrestrial mammals (see Introduction), the present study on displacement experiments in camels is an attempt to shed some new light on this topic. A recent study found that dogs may rely on magnetic compass to calibrate their homing runs when using novel routes or a scouting strategy in 33% of cases (Benediktová et al., 2020). In the absence of familiar landmarks in a forest setting where the experiments were conducted, the authors hypothesized that magnetic cues help the dogs recalibrate a path integrator mechanism based on errors accumulated during the outbound runs, so they do not impact the calculations for homebound trajectory. Previous work had shown that path integration can be reset when the animal is at home base (Alyan, 1996; Etienne et al., 2004) or at some goal point (Wehner, 2020). Thus, the use of a geomagnetic compass in the case of camels cannot be excluded, as stated above.

## MATERIALS AND METHODS

### Subjects

Camels were recruited from the Advanced Scientific Group (ASG) collection (http://asgroup.ae/; 24.436418047479116, 55.003662284309854). The ASG is primarily concerned with camel breeding and treatment. The camels never left the ASG husbandries to forage in the surrounding area. However, all of the camels had participated in few races or auctions in other areas that were at least 50 km away from ASG husbandries. The camels that participated in such events were hauled in trucks to any event's location, and when the event was finished, they were transported back to the farm. Camels begin participating in such events once they reach 3 years of age. So, a camel that is 10 years old would have been to more events than a 5-year-old camel.

The camels' collection of the ASG comes from locally bred strains and strains imported mainly from Sudan. All camels used in this study were either lactating or non-lactating females. Their ages ranged from 3- to 19 years old. In addition, all female camels used were unfamiliar with the release areas, unless stated otherwise (Table S1).

Most of the camels in the ASG collection are highly prized animals. The offspring of some of the camels we used were sold for $136,000–815,000. For minimizing stressing and accidental pathogen transfer to the animals, the experimenters, who had no experience in handling camels, had no direct contact with the experimental animals. Therefore, all contacts with the camels and all the experiments were carried out by shepherds, who had received detailed information about the experimental procedures, and who closely followed the camels during the round-trip journeys.

We worked with as many camels as we could and tried to use the same number of camels for each experimental group. However, this was not always feasible. Sometimes, we had a shortage of camels but needed to finish a specific experiment owing to an upcoming break period. In addition, owing to the COVID-19 pandemic, we were required to stop all experiments for 6 months. When we resumed work, many of the camels had been fertilized; thus, few were available. Another important factor was the availability of shepherds.

### Ethical note

All camels used in the study were handled by the ASG-assigned shepherds and personnel in adherence to their protocols and regulations. In addition, we obtained approval from the United Arab Emirates University (UAEU) animal ethics committee for this research. All camels that were used for the experiments were examined on the same day, or the next day, by the ASG’s veterinarians to ensure there were no signs of physical injuries and behavioral stress that would warrant interrupting the experiments.

### Study area

The study was conducted around the camel husbandries of the ASG, which were in the Sweihan area (24.435704979783665, 55.00368373994918; Fig. S1). The experiments were conducted at different times during the year, depending on the availability of shepherds. In summer, we recorded temperatures as high as 69°C, under sunlight, in July 2019. From November to end of March, temperatures were milder. Wind directions and speed were recorded for the days on which we conducted the experiments (Fig. S2). Two locations were selected as release points for experiment 1 (ERP and WRP; Fig. S3). The two points were north of the ASG husbandries. The area between the ASG quarters and the release points comprised sand dunes of differing heights. Sand dunes can be very wide and tall at times (Fig. S4). The area consisted of many continuous sand dunes, interspersed with flat, hard, white ground, called Seihs. On those Seihs, smaller dunes' ridges, a few meters high, also form. It is hard to tell what comes after a dune, big or small, since other dunes can connect immediately to one another, or a huge pit can follow. It is very unsafe to drive and cross dunes during daytime even for experienced desert goers. I never saw anyone crossing dunes at night. The study area has many car tracks, some that go across high dunes. It is heavily patrolled by police SUVs since it is close to a military base. Some dirt routes are found in low areas frequented by SUVs. In addition, there are four to five spots where some camel farms can be found during the winter–spring season for 3–4 months. They are mostly ephemeral farms that are abandoned soon after the breeding season. I have seen two permanent farms. The blue square farm on the map is a camel breeding center. As a general rule in desert areas, farms do not have lights at night, and this adds to the risks of driving at night, since all those farms, ephemeral and permanent, have fencing and other structures that cannot be seen unless one bumps into them. We made sure that the ASG quarters were not directly perceptible, at least not visually from the release points. A third location (3rdRP) was selected for release when the camels were hauled in trucks. The 3rdRP was a release point used to unload camels that come to a treatment center to the north of ASG facilities. It was necessary to use that release point because it had a mound that camels use to dismount from the truck. Camels, unlike horses, cannot ascend/descend from a sloping truck tailgate. The tailgate needs to be at a level with the top of the mound. The animals then descend from the mound at their own pace. The same applies for climbing onto the truck; camels need to climb the mound to a point that is in level with the truck tailgate and then walk directly into the truck (Fig. S5).

It is important here to stress that, after releasing a camel at ERP or WRP, the shepherd moved approximately 0.5 km north of the release point and waited 15–20 min before heading back to ensure that the camels were not influenced by the shepherd's path when they started moving. However, after release from the truck, the driver and the shepherd drove away from the area. After release, camels were left to move on their own, with no human presence.

### GPS Tracking

The camels used in all experiments had GPS trackers (Kingsneed TK05^®^) fitted on them. The GPS tracker was placed in a thick, multi-layered cloth pouch after losing one GPS device that camels chewed on. The pouch was hung around the camel's neck. Positional data were procured every 60 s. In one of the camel release trials (on-foot outward journey, experiment 1), three GPS trackers were fitted on the same camel to ascertain the accuracy and congruence of the tracking data, which was verified by graphing the plots. Sometimes, the fixes were absent owing to signal loss.

### Statistical analyses

Paths taken during the homeward journeys were analyzed from the moment the camels were released until they returned to the ASG quarters. The individual mean vector for each track was calculated by taking the average of the directions taken by the camel while moving from one point to the next at a speed of >1 km/h (Gagliardo et al., 2011). The mean vector distribution of each experimental group was tested for randomness using a one-sample Hotelling test (Batschelet, 1981) for the release site of the group after the homing direction had been standardized to 360°.

In addition, the EI of the tracks (the ratio between the track length and beeline distance between the release site and home) was calculated for each camel in each experimental group, except in cases when camels did not return to their home quarters. All data for each group were presented on x plots showing the second and third quartiles for each group of camels.

## Supplementary Material

Supplementary information
