# Supplementary material for: Short-range homing in camels: displacement experiments
Source: Biol Open. 2021 Aug 6;10(8):bio058850. doi: 10.1242/bio.058850 (PMC8353260; doi:10.1242/bio.058850)
Supplement: Supplementary information [file biolopen-10-058850-s1.pdf]

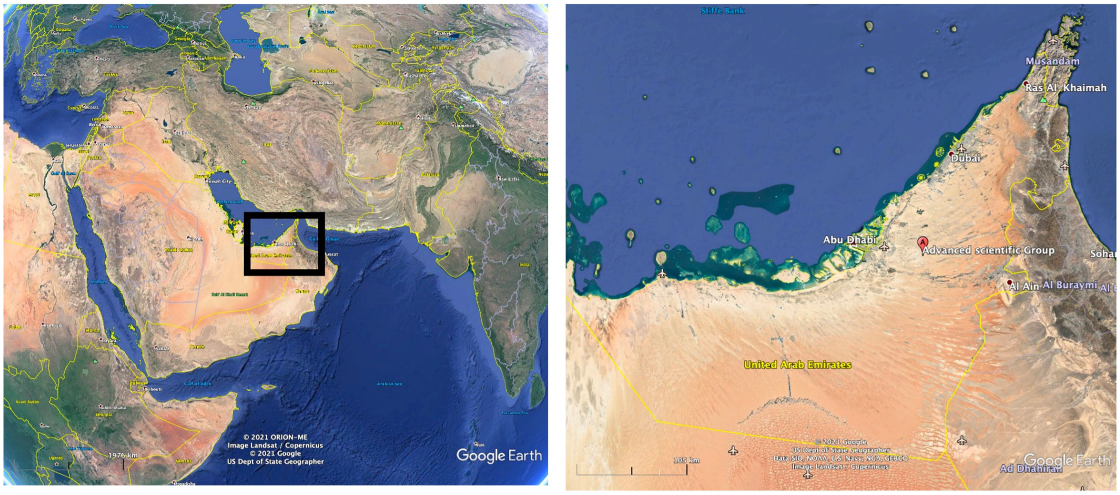

Fig. S1. A map showing the United Arab Emirates (UAE, inside the black box on the left map), and the location of the study site within the UAE, indicated by the red balloon, on the right map.

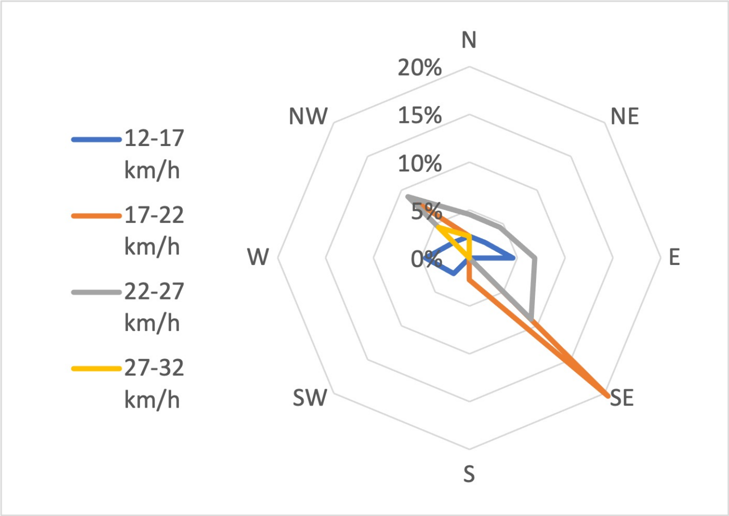

Fig. S2. Wind rose representation of prevailing winds during experimental days. The prevailing winds came from the NW and SE directions.

Biology Open • Supplementary information

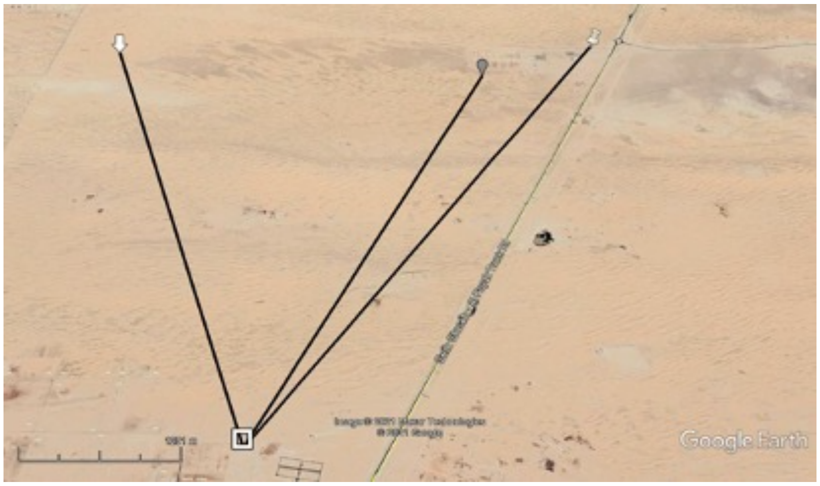

Fig. S3. The camels were taken from the ASG husbandries, indicated by the white square, and released in one of three points: East release point (ERP) indicated by grey balloon, west release point (WRP) indicated by white arrow, and third release point (3rdRP) used for camels transported in trucks (white pin). The black lines show the beeline path between each release point and the starting point for all camels.

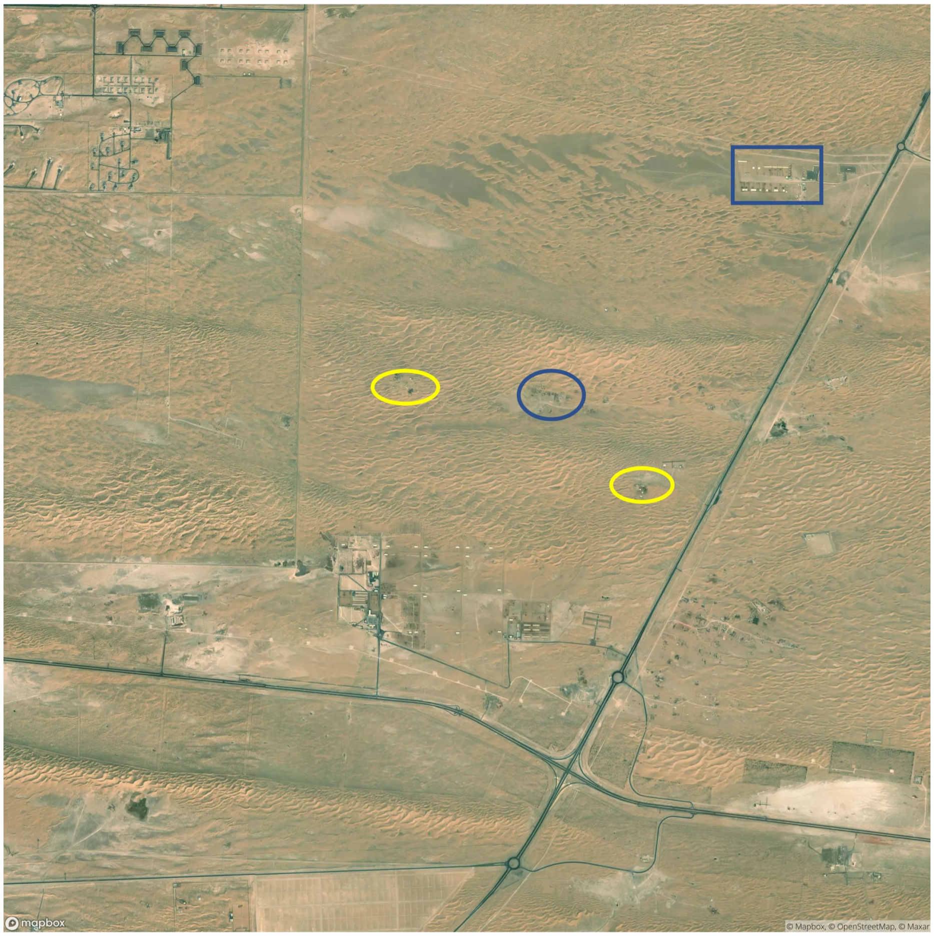

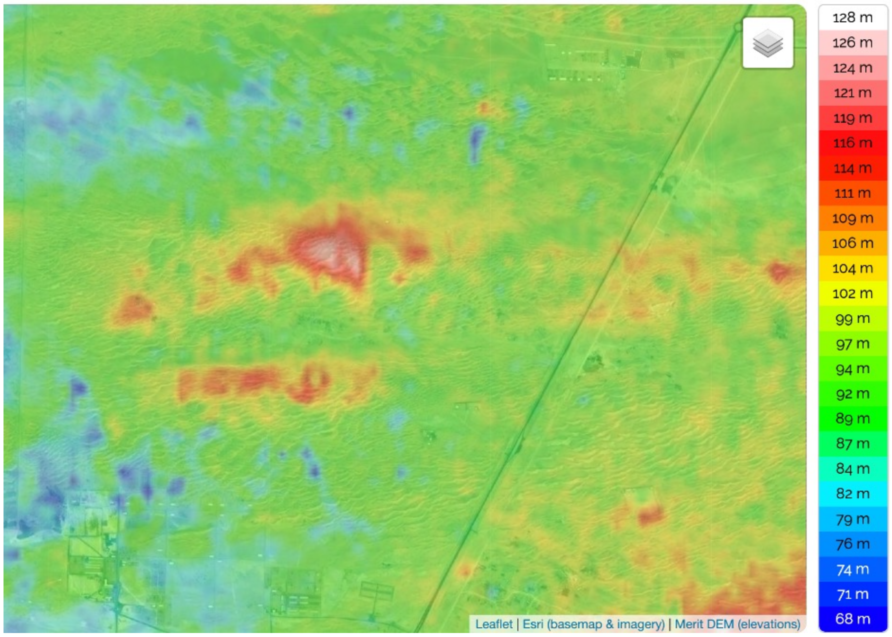

Fig. S4. Two images to clarify the topography of the experimental area. The top was taken from mapcarta.com, and the bottom one from topographicmap.com. In the top map, two permanent farms can be seen (blue circle and rectangle) and two ephemeral farms (two yellow ovals).

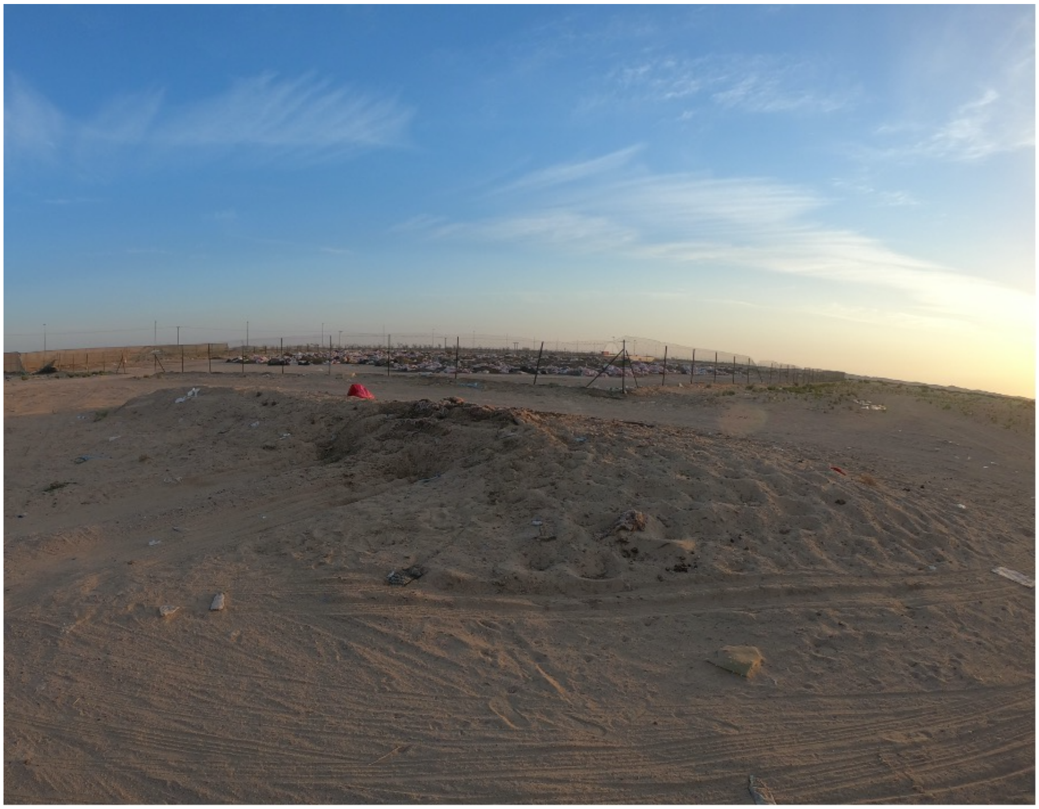

Fig. S5. Mound used to upload/unload camels from trucks.

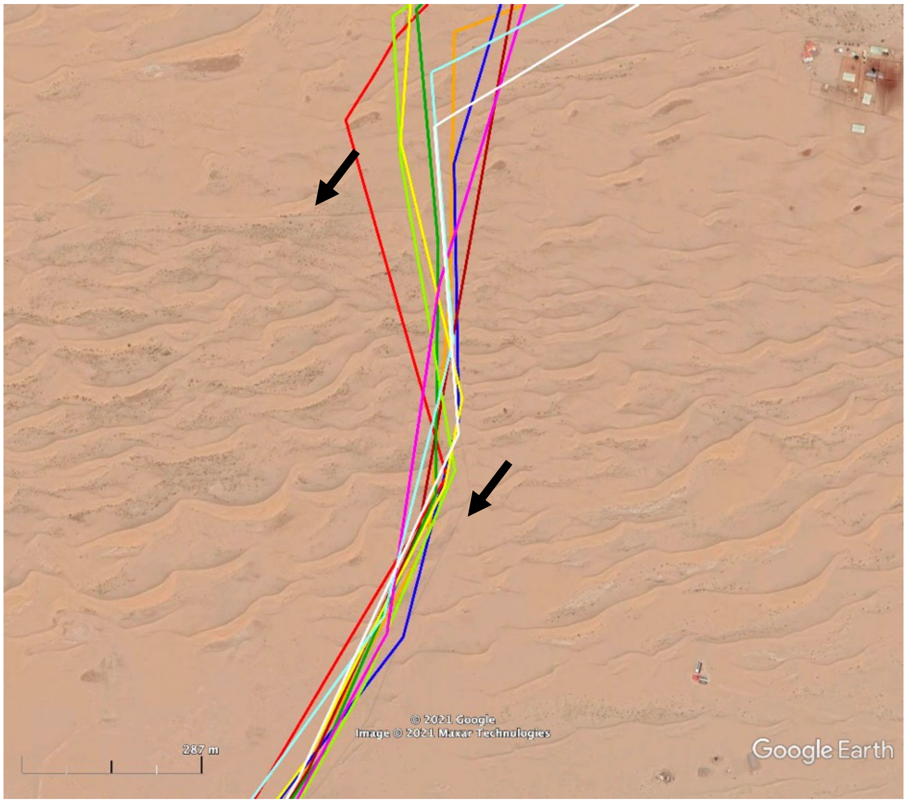

Fig. S6. This figure shows convergence of all camel tracks to a narrow area along the homeward path. A small route can be seen along the path (black arrows), which might be used by the camels as they navigate towards their quarters.

Biology Open • Supplementary information

Table S1. Basic data of all camels that were used in the different experimental groups. Shaded cells indicate day-time releases. Wind direction 0/360 is set to be North.

	Date	Time of day	Age (yrs)	release point	Nursing	Wind speed km/h	Wind Dir [°]
**Experiment 1: On Foot 2019-naïve**	2-Jul	day-time	6	east	lactating	19	144
	2-Jul	night-time	7	east	nonlactating	19	2
	3-Jul	night-time	7	west	nonlactating	26	329
	4-Jul	day-time	3	west	lactating	25	125
	5-Jul	night-time	7	east	lactating	22	320
	5-Jul	day-time	5	east	nonlactating	22	140
	6-Jul	day-time	6	west	nonlactating	17	157
	17-Jul	night-time	9	east	nonlactating	19	130
	18-Jul	night-time	3	east	nonlactating	36	325
	20-Jul	day-time	7	east	nonlactating	23	66
**Experiment 2: In Truck 2019-2020-naïve**	22-Dec	night-time	4	3rdRP	lactating	13	240
	23-Dec	night-time	3	3rdRP	nonlactating	19	305
	24-Dec	night-time	12	3rdRP	nonlactating	15	323
	24-Dec	day-time	9	3rdRP	lactating	15	30
	25-Dec	night-time	15	3rdRP	lactating	15	259
	3-Jan	night-time	5	3rdRP	nonlactating	13	11
	21-Sep	night-time	5	3rdRP	nonlactating	30	302
	21-Sep	day-time	10	3rdRP	lactating	30	359
	22-Sep	night-time	7	3rdRP	lactating	35	317
	22-Sep	day-time	12	3rdRP	lactating	35	58
	15-Feb	night-time	3	3rdRP	nonlactating	20	304

**Experiment 3: In Truck-covered up-2020-naïve**	16-Feb	night-time	9	3rdRP	lactating	12	81
	18-Feb	day-time	12	3rdRP	lactating	24	128
	18-Feb	night-time	6	3rdRP	nonlactating	24	326
	19-Feb	day-time	3	3rdRP	lactating	14	94
	19-Feb	night-time	7	3rdRP	nonlactating	14	288
	20-Feb	day-time	16	3rdRP	lactating	19	117
	20-Feb	night-time	5	3rdRP	nonlactating	19	194
	25-Feb	day-time	13	3rdRP	nonlactating	19	120
	30-Mar	day-time	7	3rdRP	nonlactating	23	75
**Experiment 4: In Truck-disorientation-2020-naïve**	3-Oct	night-time	15	3rdRP	nonlactating	21	308
	6-Oct	day-time	10	3rdRP	nonlactating	23	92
	9-Oct	day-time	19	3rdRP	nonlactating	22	111
	10-Oct	night-time	5	3rdRP	lactating	27	323
	12-Oct	day-time	5	3rdRP	nonlactating	24	354
**Experiment 5: In Truck-straight-2020- repeat**	26-Oct	day-time	10	3rdRP	nonlactating	21	132
	27-Oct	day-time	19	3rdRP	nonlactating	22	122
	29-Oct	day-time	5	3rdRP	lactating	23	54
	31-Oct	night-time	15	3rdRP	nonlactating	21	142
**Experiment 6: In Truck-disorientation-2020-repeat**	14-Oct	day-time	9	3rdRP	lactating	20	129
	17-Oct	night-time	12	3rdRP	nonlactating	22	334
	18-Oct	night-time	12	3rdRP	lactating	25	360

Table S2. Duration in minutes of the outward and homeward legs for camels in Experiment 1, 2 and 4.

For Experiment 2, all outward journeys used a truck to transport camels, so the times were all the same.

For Experiment 4, only the outward trips' times are displayed.

Experiment 1	
	homeward
July 2 day	39
July 2 night	44
July 3 night	53
July 4 day	60
July 5 day	32
July 5 night	44
July 6 day	67
July 17 night	33
July 18 night	32
July 20 day	29

Experiment 2	
	homeward
Dec 22 night	52
Dec 23 night	48
Dec 24 day	49
Dec 24 night	42
Dec 25 night	43
Jan 3 night	68
Sep 21 day	33
Sep 21 night	46
Sep 22 day	31
Sep 22 night	39

Experiment 4	
	outward
Oct 3 night	100
Oct 6 day	120
Oct 9 day	80
Oct 10 night	110
Oct 12 night	113
